# Plasmin Cascade Mediates Thrombotic Events in SARS-CoV-2 Infection via Complement and Platelet-Activating Systems

**DOI:** 10.1109/OJEMB.2020.3014798

**Published:** 2020-08-06

**Authors:** Kavitha Mukund, Kalai Mathee, Shankar Subramaniam

**Affiliations:** ^1^ Department of BioengineeringUniversity of California San Diego 8784 La Jolla CA 92093 USA; ^2^ Department of Human and Molecular GeneticsHerbert Wertheim College of Medicine 158263 Miami FL 33199 USA; ^3^ Biomolecular Sciences InstituteFlorida International University 5450 Miami FL 33199 USA; ^4^ Department of Cellular and Molecular MedicineUniversity of California San Diego 8784 La Jolla CA 92093 USA; ^5^ Department of Computer Science and EngineeringUniversity of California San Diego 8784 La Jolla CA 92093 USA

**Keywords:** COVID-19, plasminogen activators, thrombosis, complement system, neutrophil extracellular traps (NETs)

## Abstract

*Objective:* Recently emerged beta-coronavirus SARS-CoV-2, has resulted in the current pandemic designated COVID-19. COVID-19 manifests as severe illness exhibiting systemic inflammatory response syndrome, acute respiratory distress syndrome (ARDS), thrombotic events, and shock, exacerbated further by co-morbidities and age. Recent clinical evidence suggests that the development of ARDS and subsequent pulmonary failure result from a complex interplay between cell types (endothelial, epithelial and immune) within the lung promoting inflammatory infiltration and a pro-coagulative state. How the complex molecular events mediated by SARS-CoV-2 in infected lung epithelial cells lead to thrombosis and pulmonary failure, is yet to be fully understood. *Methods:* We address these questions here, using publicly available transcriptomic data in the context of lung epithelia affected by SARS-CoV-2 and other respiratory infections, in vitro. We then extend our results to the understanding of in vivo lung, using a publicly available COVID-19 lung transcriptomic study. *Results and Conclusions:* Our analysis indicates that there exists a complex interplay between the fibrinolytic system particularly plasmin, and the complement and platelet-activating systems upon SARS-CoV-2 infection, with a potential for therapeutic intervention.

## I. Introduction

Recent studies [1], [2] have rapidly provided molecular insights into the pathogenicity of the SARS-CoV-2, mainly at the level of genomic, structural, and functional aspects of viral-host interactions. Further, studies have identified key pathophysiological and molecular events associated with infection pathogenesis and progression of COVID-19 including thrombocytopenia, lymphopenia, eosinophilia, and elevated lactate dehydrogenase and fibrinogen [3], [4]. The molecular findings have included a delayed interferon (IFN) response type I and III [5], [6] with a concomitant increase of proinflammatory cytokines (namely IL-17, IL-6 and/or IL-1B) [7] leading to a “cytokine” storm coupled with the depletion of markers for platelets, natural killer cells, and dysregulation of CD4+ and B-cell lymphocyte populations [8]. In contrast to other respiratory viral infections (e.g., refs [9]–[12]), SARS-CoV-2 can efficiently replicate in cells of various tissues that express angiotensin-converting enzyme 2 (ACE2) and host serine protease (TMPRSS2) [13], [14], thus contributing to the increased transmissibility and lower-lung pathogenicity in humans [15]. This observation led us to explore mechanisms unique to SARS-CoV-2 infection of lung epithelial cells and cause thrombotic events.

## II. Results

### A. Comparing the SARS-CoV-2 Transcriptomic Profile With Other Upper-Respiratory Tract Infections

**Figure 1.**
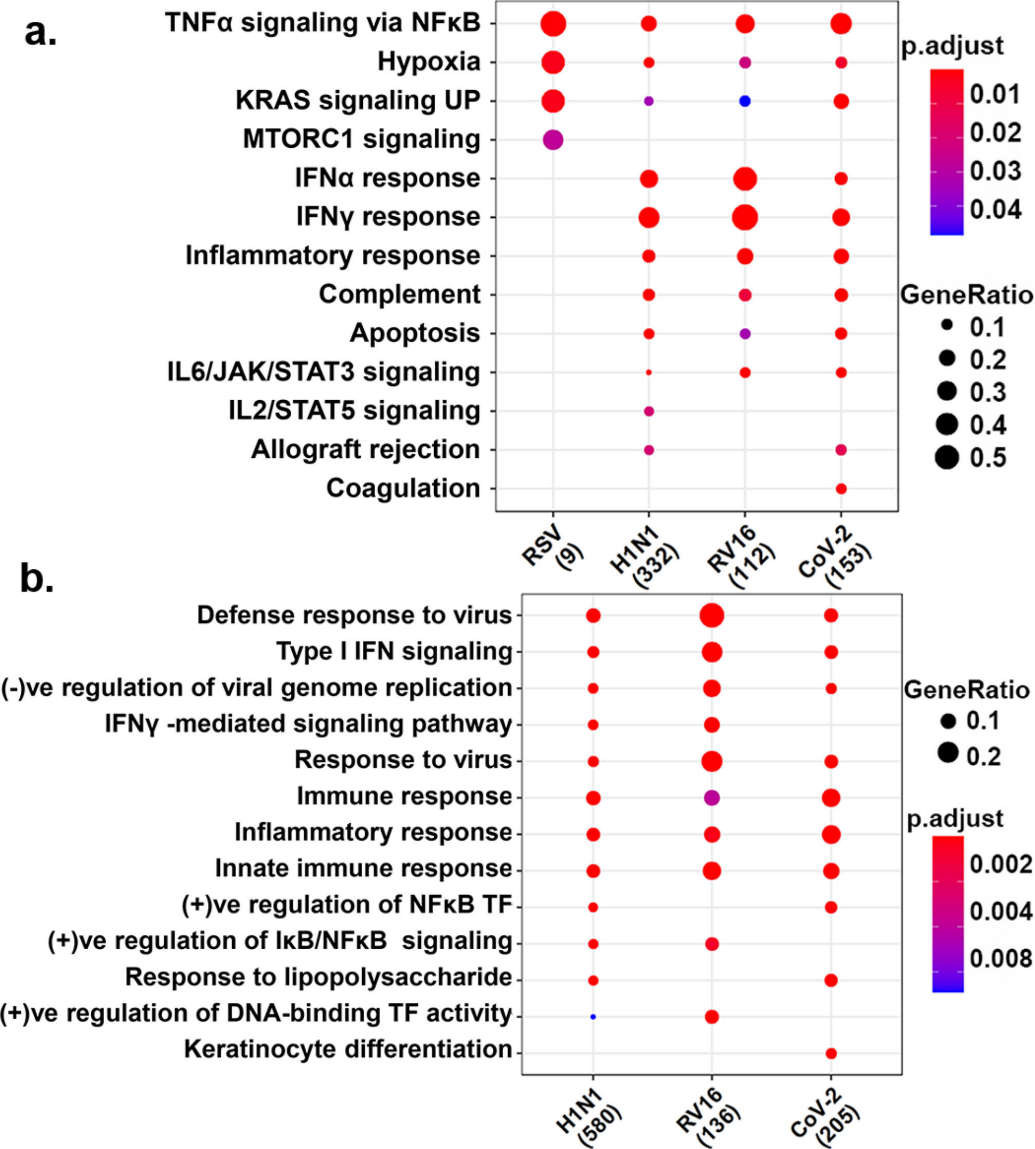
Functional enrichment of upregulated genes in upper respiratory tract infections- Functional enrichment of upregulated genes in upper respiratory tract infections- Functional enrichment of genes upregulated upon infection with Rhinovirus (RV16), respiratory syncytial virus (RSV), influenza (H1N1) and SARS-CoV-2 (CoV-2) were identified using (a). mSigDB's hallmark gene sets and (b). Gene ontology's (GO) biological process enrichment. No significant GO enrichment was identified for RSV and hence not included in b. Enrichment illustrated the biological functions common and unique to the different infections. Gene ratios are indicated by the dots’ size and the adjusted p- value is by the color scale indicated.

In this study, we utilized publicly available RNA-sequencing data [5] (GSE100457) from Normal Human Bronchial Epithelial (NHBE) cell lines infected with SARS-CoV-2 (henceforth referred to as the CoV-2 dataset) and compared it with lung epithelial cells infected with other respiratory infections, namely, respiratory syncytial virus (RSV), influenza (H1N1), and rhinovirus (RV16) using stringent study inclusion criterion (see Methods). Differentially expressed genes (DEGs) were called for each infection (with respect to their respective controls) using limma/voom, at an adjusted p-value < 0.05 (see Methods). We identified a total of 339, 27, 1781, and 208 DEGs in CoV-2, RSV, H1N1 and RV16 datasets, respectively, with a more significant proportion of genes upregulated, in all cases, in response to infection (Supplementary Table 1). Our analysis indicated a significant over-representation of functional categories associated with immune response, across all infections (among upregulated genes) (Fig. 1). The categories included, “response to virus”, “TNFα signaling via NFκB”, “interferon response” (types I/α, II/γ), and “apoptosis”. However, “coagulation” and “keratinocyte differentiation pathways” were among the top pathways to be uniquely enriched within the CoV-2 dataset. Genes including *ANXA1*, *C1S*, *C3*, *CFB*, *F3*, *ITGB3*, *MAFF*, *MMP1*, *MMP9*, *PDGFB*, *PLAT*, *PLAU*, *SERPINB2*, *TFPI2* associated with “coagulation” and *S100A7, ANXA1, TGM1, IVL,* and small proline-rich proteins *SPRR2A, SPRR2E, SPRR2D,* associated with the “keratinocyte differentiation pathway” were among the 161 genes that were “uniquely” significantly upregulated within the CoV-2 dataset (Fig. S1). Keratinocytes are known to be associated with epithelial cell repair.

### B. Identifying functional mechanisms unique to SARS-CoV-2 infections

**Figure 2.**
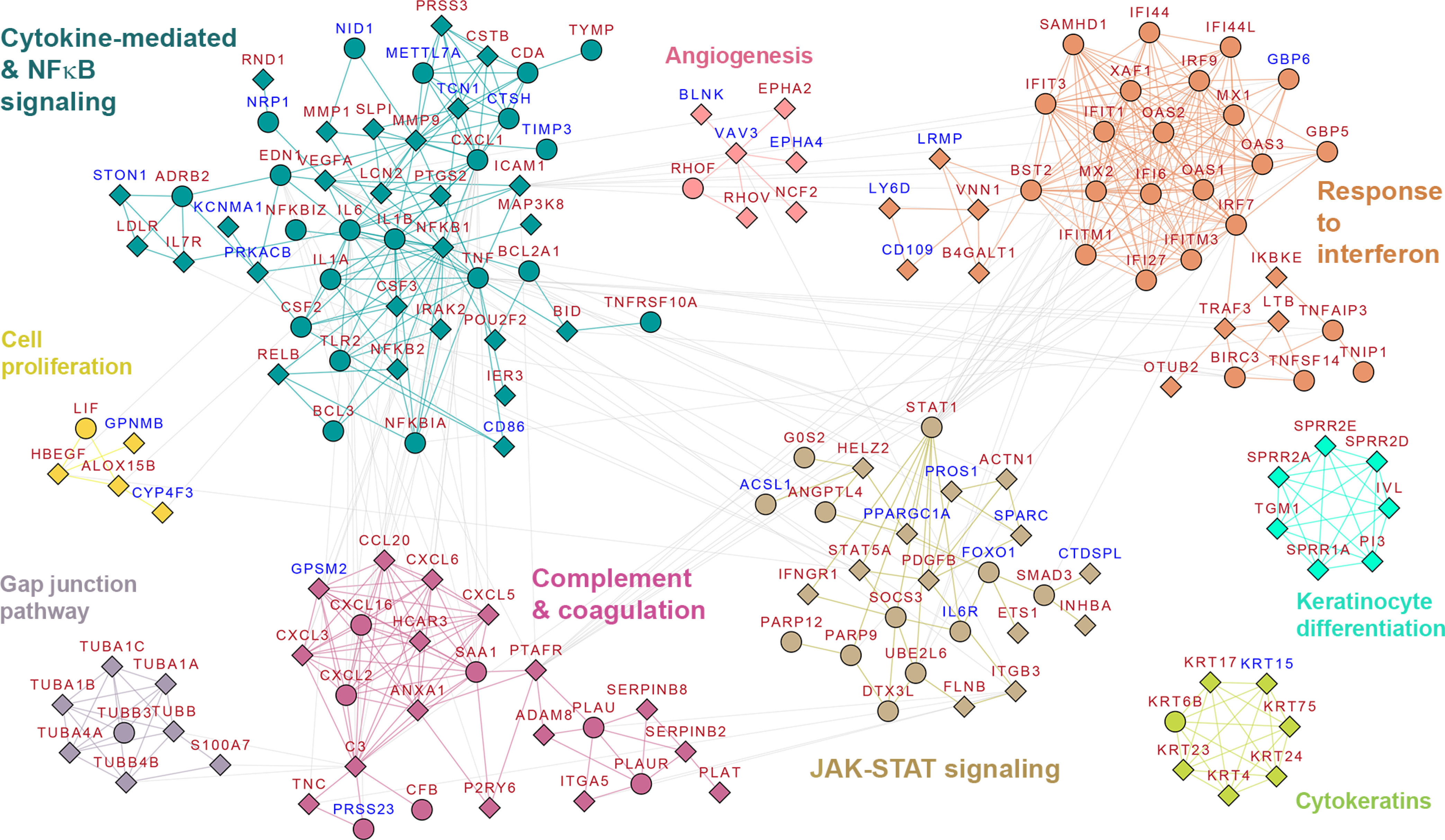
Protein interaction network for SARS-CoV-2 infection- The protein-protein interaction network extracted for genes differentially regulated (DEGs) in CoV-2 dataset (see Methods) is shown here. Clustering identified nine functional modules for further analysis. The modules functionally corresponded with i. Response to interferon, ii. Cytokine-mediated & NFkB signaling iii. Complement & coagulation iv. JAK-STAT signaling v. Cell proliferation vi. Gap junction pathway vii. Cytokeratins viii. Keratinocyte differentiation and ix. Angiogenesis. Red node labels indicate upregulated genes and blue node labels indicates downregulated genes. Diamond node shapes indicate DEGs identified only within CoV-2 while circle indicate DEGs identified in more than one upper respiratory tract infection.

We posed the question if analyzing the molecular cascades associated uniquely with SARS-CoV-2, identified above, could translate to findings that are relevant to thrombotic outcomes seen in severe/acute COVID-19 patients [16]. To relate the clinical and molecular phenotypes associated with SARS-CoV-2 infection, we generated a protein interaction map (strength > 0.85) from DEGs identified within the CoV-2 dataset (see Methods). We annotated this network with data from the comparative analysis for the three other infections (RSV, RV16, and H1N1). We subsequently clustered this network, to identify “functional modules” relevant to the pathology of SARS-CoV-2 infection. Our clustering identified nine modules with five smaller functional modules containing genes associated uniquely with SARS-CoV-2, involving angiogenesis, tubulins, keratinocytes, and small proline-rich proteins (Fig. 2). Three of the four remaining modules were mainly associated with immune response. These functional modules included (i) genes associated with response to IFN type I/III such as *IFITM1*, *IFITM3*, *MX1*, *MX2*, *OAS1*, *OAS2*, *IFIT1*, *IFIT3*, and *IRF9*; (ii) Activation of the IL-17 pro-inflammatory cascades (*IL6*, *IL1B*, and *CXCL1*) via the TNFα and NFκB signaling cascades (*TNF*, *CSF2*, *CSF3*, *NFKB1*, *NFKB2*, *RELB*, and *IRAK2*) (iii) Signaling cascades downstream of interleukin response including JAK-STAT containing genes such as *IL6R*, *STAT1*, SOCS3, *IFNGR1,* and *PDGFB*. Interestingly, though these modules were broadly representative of vital immune mechanisms seen in all respiratory infections [17], [18], they also highlighted specific mechanisms unique to SARS-CoV-2. For example, subversion of innate immune response is inherent to cytopathic pathogens like SARS-CoV (a related beta coronavirus), which causes an increased release of pyroptosis products (e.g. IL-1B), inducing acute inflammatory responses. The antagonism of an interferon response by SARS-CoV viral proteins has been suggested to occur at multiple stages of the interferon and NFKB signaling cascades, through multiple mechanisms, including IKBKE and TRAF3 regulation, affecting downstream *STAT1* associated signaling [19]. It is possible that, in the SARS-CoV-2 infection, the observed upregulation of *IKBKE*, *TRAF3, NFKB1, NFKB2, RELB, and IRAK2* at 24hpi could be a consequence of similar mechanisms [2] The fourth large module, contained genes specifically activated within the CoV-2 dataset, involved in fibrinolysis and plasminogen activation cascades (*PLAT, PLAU, PLAUR*, and *SERPINB2*), the complement activation cascade including *C3, CSB, CXCL5*, and platelet aggregation (platelet activation factor receptor- *PTAFR* (Fig. 2)). This module was particularly noteworthy given the enrichment results (Fig. 1A) and clinical findings of COVID-19 [16]. It is analyzed further in the following sections.

### C. Translating Functional Mechanisms Mediated by SARS-CoV-2 Infections to COVID-19

Plasminogen is the precursor of the serine protease *plasmin*, a vital component of the fibrinolytic system, essential for ensuring immune cell infiltration and cytokine production [20]. Plasminogen can be activated to plasmin by two serine peptidases, the tissue tissue-specific (T-PA) and urokinase (U-PA) plasminogen activators encoded by *PLAT* and *PLAU*, respectively. It has been previously reported for SARS-CoV and influenza infections that dysregulation of the urokinase pathway including U-PA(*PLAU*) and its inhibitor PAI-1*(SERPINE1)* might contribute to the severity of lung disease by altering the dynamics of fibrin breakdown and intra-alveolar fibrin levels and subsequently inflammation [21], [22]. We observed a similar dynamic with SARS-CoV-2 infection albeit through activation of tissue plasminogen, *PLAT,* and the inhibitor *SERPINB2*. T-PA (which is triggered when bound to fibrin) and *F3* (tissue factor, activated in CoV-2) levels are known to correlate with d-dimer levels [23]. D-dimer, a product of fibrin degradation by plasmin, is elevated in patients with COVID-19 and has been identified as a marker for disseminated intravascular coagulopathy and a worse patient prognosis [24]. These findings indicate increased fibrinolytic activity, specifically via T-PA (*PLAT)* activation, in SARS-CoV-2 infections.

The outcome of any viral infection is mediated through a complex interplay between viral and host proteins, which allow for a coordinated innate immune response. Plasminogen inhibitor (PAI)-1 (*SERPINE1*) has been reported to function as an anti-viral factor capable of inhibiting extracellular maturation of influenza particles, specifically through their action on *TMPRSS2* [25]. A similar mechanism involving PAI-2 (*SERPINB2)* likely exists in SARS-CoV-2 infections. Additionally, T-PA (*PLAT*) has been reported to interact with *ORF8* protein of SARS-CoV-2 virus [2]; however, its consequence has not yet been elucidated. We hypothesize that if this interaction titrates out T-PA, it is likely to result in reduced levels of plasmin, which could contribute to downstream thrombosis events, as observed in COVID-19 patients [16]. Increased expression of *PLAT* validated this. Additionally, increased concentration of *SERPINB2* is also likely to reduce plasmin levels leading to thrombosis. The unique regulation of *SERPINB2* and *PLAT* in SARS-CoV-2 infections and their subsequent effect on viral- host interaction dynamics is worthy of further investigation.

Matrix metalloproteinases (MMPs) are a family of proteolytic enzymes that facilitate leukocyte infiltration by breaking down the extracellular matrix (ECM) and basement membrane. The genes *MMP1* and *MMP9* (both upregulated specifically within the CoV-2 dataset, see Fig. 2) can also be activated by plasmin *in vivo* and *in vitro* [26]. Additionally, the degranulation of neutrophils by T-PA has been indicated as a source for the increase of matrix metalloproteinase 9 (MMP-9) [27]. Given the observed increase in MMPs, and their role in facilitating lung inflammation in ARDS (by enabling neutrophil migration and ECM breakdown) [28], it will be essential to evaluate the impact of T-PA treatments [29] on pulmonary remodeling (via MMPs) in patients with severe/acute COVID-19.

We identified significant upregulation of several genes encoding components of the complement system, including *C3, CFB,* and *C1S*, uniquely within the CoV-2 dataset, in contrast to other upper respiratory tract infections (at 24 hpi). The complement pathway is an integral part of the innate immune response and is involved in immunosurveillance for pathogen clearance (bacterial and many viral) [30]. It traditionally serves as a vital link between the innate and the adaptive immunity, mainly driving pro-inflammatory cascades. Several chemokines/chemoattractants (*CXCL5, CXCL6*, *CXCL3*, and *CCL20*) identified within this cluster can also be activated by signaling events precipitated by complement activation [31], p. 5]. Overstimulation of these chemokines, particularly CXCL5, is known to cause destructive inflammatory lung conditions in specific pathogenic models of lung disease [32].

Plasmin is known to activate complement cascade independently of established pathways (alternate, lectin, and classical pathways) by cleaving *C3* and *C5* to functional anaphylatoxins C3a and C5a, *in vitro* and animal model studies [33]. Furthermore, there is increasing evidence for the role of complement in coagulation. C3 binds fibrinogen and fibrin with high affinity and prolonging fibrinolysis in a concentration-dependent manner [34]. Studies in animal models of thrombosis have identified a plasmin-driven C5a generation capable of driving procoagulant cascades [35]. Moreover, the presence of terminal complement components including C5b-9 (membrane attack complex/MAC), C4d, and mannose-binding lectin (MBL)-associated serine protease (*MASP2*) within the pulmonary microvasculature and purpuric skin lesions of deceased COVID-19 patients [36]. MAC has been additionally suggested to promote neutrophil adhesion via platelet activating factor (PAF)-mediated mechanisms [37]. We observe a unique activation of the PAF receptor (PAFR, encoded by the gene *PTAFR*) within the complement and coagulation cluster (Fig. 2). PAF is a potent activator of platelet aggregation as well as immune cell types including macrophages and neutrophils [38]. PAF binding to PAFR activates several intracellular signaling events including complement activation [39], and plays pathophysiological roles in molecular mechanisms underlying anaphylaxis, bronchial asthma, cystic fibrosis [40], and endotoxin shock/sepsis [41]. It is a crucial mediator of systemic and pulmonary hemodynamic changes, additionally contributing to pulmonary edema [42], [43] fitting the COVID-19 lung injury model [44]. Immune infiltration primed by interaction between complement system and PAF has been reported previously. Specifically, C5a and PAF together can induce massive eosinophil transmigration [45]. Eosinophils release leukotrienes and lipids, which can cause further epithelial damage and airflow obstruction [46]. Leukotrienes, C5a, and PAF are also potent chemo-attractants of polymorphonuclear leukocytes (neutrophils or PMNs), which can further activate neutrophil extracellular traps (NETs) leading to pulmonary damage seen in COVID-19 patients [47]. Given the ubiquity of SARS-CoV-2 receptors and its ability to infect a broad range of cell types [13], analysis of *PTAFR* activation patterns in infected tissues (including vascular endothelial cells) could provide further insights into increased platelet aggregation (via PAF signaling) and the risk for arterial thrombosis seen in severe/acute COVID-19 patients [16]. Vitamin-D is known to attenuate *ICAM1* and *PTAFR* expression and subsequently NFκB mediated inflammation, in rhinovirus infections [48]. ICAM1 is a crucial mediator of inflammation and is suggested to mediate eosinophil adhesion to airway epithelium [49]. Similar mechanisms can underly the recent reports on reduced risk for COVID-19 infections after Vitamin-D supplementation, and provide evidence for PAF/PAFR mediated signaling in the progression of COVID-19 [50].

## III. Discussion

The significant enrichment of mechanisms identified above were indicative of active epithelial remodeling and provided evidence for a plasmin-mediated complement activation within SARS-CoV-2 in contrast to other respiratory infections. We also identified mechanisms that might contribute to increased inflammation via immune infiltration and platelet aggregation, primed by the interactions between the complement system, plasmin and the PAF/PAFR. Our findings further the understanding of modulation of hemostatic factors, affecting the mechanics of circulating fibrin and fibrin breakdown products [3] and complement activation, underlying acute thrombotic events including arterial thrombosis in COVID-19 patients [16].

**Figure 3.**
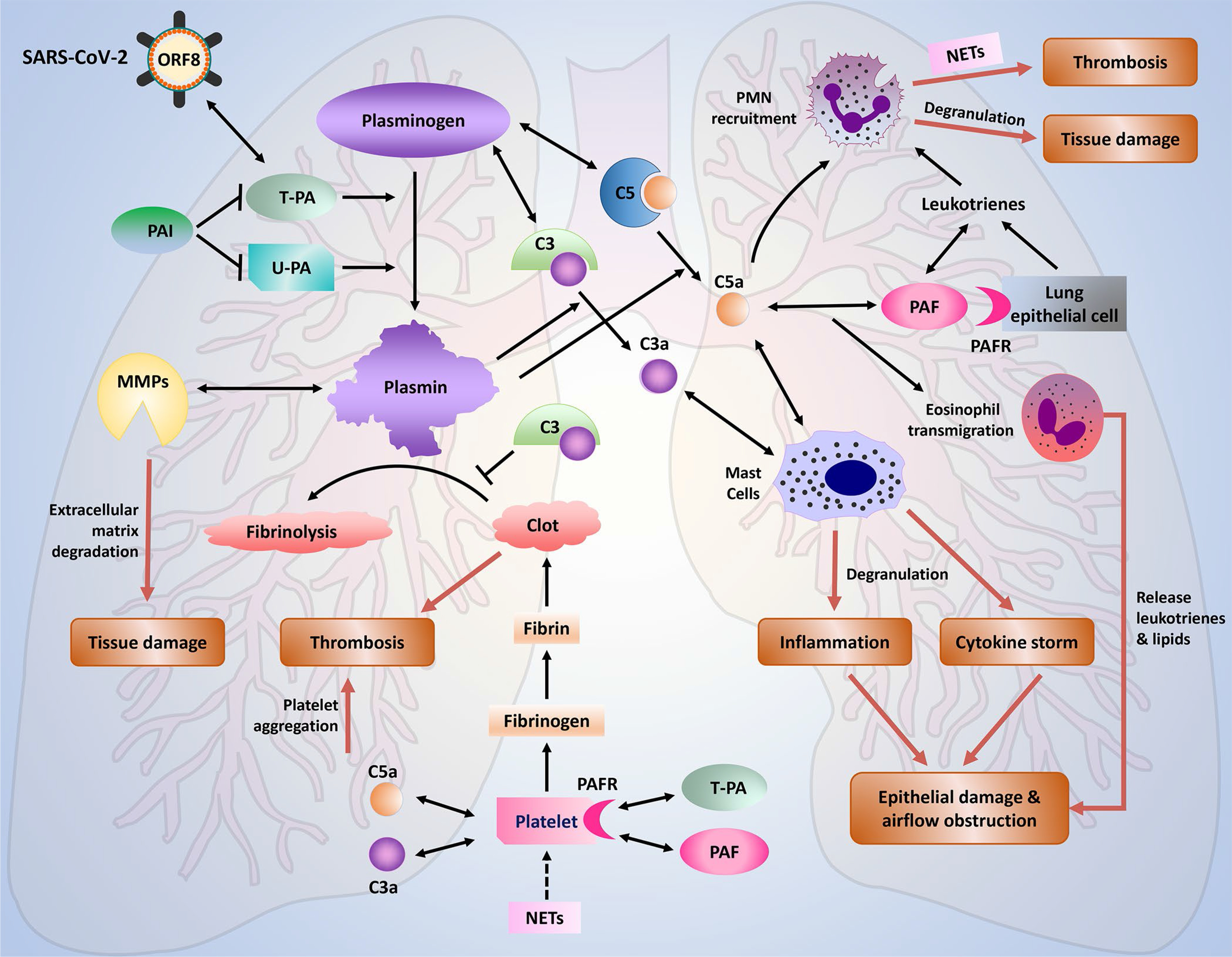
A schematic representation of the crosstalk between plasmin, complement, and platelet-activating systems in SARS-CoV-2 infection. Plasminogen conversion is mediated by either by tissue-specific plasminogen activator (T-PA) or urokinase plasminogen activator (U-PA), whose activities can be inhibited by the inhibitors, PAI-1 (SERPINE1) or PAI-2 (SERPINE2B). The conversion of plasminogen to active plasmin is critical for blood clot breakdown. Failure to breakdown the clots (fibrinolysis) leads to thrombosis. Fibrinolysis can be inhibited by complement component C3. The complement components C3 and C5 can be activated by plasmin in addition the classical, lectin, and alternative pathways. The anaphylatoxins, C3a and C5a, interact and stimulate mast cells to degranulate, releasing histamine, cytokines, granulocyte-macrophage colony-stimulating factor, leukotrienes, heparin, and several proteases that damage the tissues. Overstimulation of complement cascade leads to inflammation, cytokine storm resulting in epithelial damage, and airflow obstruction that manifests as the acute respiratory distress syndrome (ARDS). Also, C5a and Leukotriene-bound PAF are potent attractants of polymorphonuclear leukocytes (PMNs) to the site of damage. The recruited PMNs can release microbiocidal molecules and form the neutrophil extracellular traps (NETs). NETs are proinflammatory and promote tissue damage, thrombus formation, and activate platelets. PAF is also suggested to trigger pulmonary edema in models of acute lung injury. Degradation of the basement membrane/ ECM promote by matrix metalloproteinases further promote immune cell infiltration and tissue damage. NETs, tissue injury, platelets-activating factors (PAF), T-PA (if overexpressed), C3a and C5a activate platelets to aggregate on a fibrin scaffold to form clot. Clots and tissue injury lead to airflow obstruction that manifests as acute respiratory distress syndrome (ARDS). Double arrows indicate association.

In addition, analysis of DEGs from transcriptomic measurements in postmortem lung tissue from COVID-19 patients provided a partial insight into the mechanisms associated with virus-altered epithelial function. Functional analysis of DEGs identified with COVID-19 exhibited an upregulation of similar immune cascades as those identified within the CoV-2 dataset (Figs. S2 and S3). Significant correlation was also identified for foldchanges between DEGs commonly regulated between COVID-19 lung and CoV-2 dataset (rho = 0.34). We present our current view of the crosstalk between plasminogen, complement and platelet-activating systems in SARS-CoV-2 infection in Fig. 3.

We are cognizant that we cannot wholly ascribe all mechanisms observed within a single time point (24 hpi) as contributing to observed clinical pathology of COVID-19, given the limited availability of relevant publicly available human *in vitro* data. However, our analysis highlighted SARS-CoV-2 driven response of healthy lung epithelial cells and its ability to incite a misdirected cascade of interactions between the coagulation, complement activation, and proteins involved in the fibrinolytic system. Using current knowledge of these three systems and the analysis of the data from *in vitro* model systems we infer the consequences of this misdirected network of interactions, namely, coagulation, inflammation, and vascular leakage/pulmonary edema in the COVID-19 lung. Detailed *in vivo* and *in vitro* studies will be required to better understand the viral-host dynamics with respect to T-PA (*PLAT)* and PAFR (*PTAFR)* activation and the subsequent systemic inflammatory responses, in symptomatic and asymptomatic COVID-19 populations.

## IV. Conclusion

Our analysis hypothesizes that managing the equilibrium between the complement, coagulation and platelet-activating components can determine the overall biological activity and the outcome of a disease severity in COVID-19. An investigation into the efficacy of complement inhibitors (such as Eculizumab [51] or Compstatin [52]), and plasminogen activators specifically for T-PA, combined with Vitamin-D supplementation to alleviate symptoms in patients with severe/acute COVID-19, warrant exploration.

## V. Materials and Methods

### A. Data acquisition

We analyzed the data recently published by Blanco-Melo *et al.* [5] to gain mechanistic insights into the pathogenicity of SARS-CoV-2. The published dataset contained multiple cell lines treated with SARS-CoV-2 including NHBE, Calu-3, A529 (with and without exogenous expression of ACE2) in addition to COVID-19 lung and normal tissue samples (available via GSE147507). Histogram of counts within normal and diseased lung samples indicated that one COVID-19 lung sample (Covid Lung 2, Fig. S4) is a likely an outlier and was ignored from further analysis. Based on the sample clustering results of the raw counts (Fig. S2.B), we limited our analysis to NHBE/ normal human bronchial epithelial cell lines (hence forth referred to as the CoV-2 dataset). All available series (GSE) in GEO were extracted from the gene expression omnibus with key words- “SARS-CoV”, “MERS-CoV”, “RSV” or “respiratory syncytial virus”, “Influenza” and “Rhinovirus” for respiratory cell lines (NHBE or BEAS-2B). Since the CoV-2 transcriptional data was processed at 24 hpi (hours post infection), we chose to compare only those infections which had cell-lines at 24 hpi yielding the following series GSE3397, GSE71766, GSE100504, GSE81909, GSE27973, and GSE28904. To limit the impact of sequencing technologies, we utilized only Affymetrix, the technology with the most coverage among the series considered. Applying the above stringent inclusion criteria yielded 3 GEO series GSE3397 [53] (RSV), GSE71766 [54] (Influenza/H1N1 and Rhinovirus/RV16) and GSE27973 [55] (Rhinovirus/RV16). We, however, did not find studies on related beta-coronaviruses in NHBE/BEAS-2B cell lines which matched our inclusion criterion.

### B. Differential expression analysis

For the sake of reproducibility, we called differentially expressed genes (DEGs) at adjusted p.value <0.05, using GEO2R for GSE3397, GSE71766 and GSE27973 comparing infected cells with their respective mocks at 24 hpi only. The DEGs called for GSE27973 were a subset of GSE71766 RV16 comparisons and subsequently ignored. Since these were microarray studies, we aggregated probes which were significantly differentially expressed, to gene names and calculated a mean fold change (Supplementary Table 1) and considered these for all comparisons outlined in the manuscript. We utilized the same pipeline implemented in GEO2R to call DEGs from CoV-2 data (using the limma-voom pipeline in R). Low counts were filtered using the “FilterByExpression” feature available through the edgeR package. “topTable” was used to extract all samples under our significance threshold of p.adj <0.05. We would additionally like to point out that we reanalyzed the CoV-2 data using the DESeq2 protocol as described in the original publication consistently identified similar number of DEGs as detected through limma-voom. For the comparision of COVID-19 lung biopsy sample against two healthy tissue biopsies, we utilized the exact T-test via “edgeR” to establish significance and extracted the significant genes via “topTags”.

### C. Protein network construction

The human protein-protein interaction network (PPIN) was downloaded from STRINGdb (v 11.0) [56] for a combined edge strength of >0.85. We extracted a SARS-CoV-2 infection relevant subnetwork from this PPIN using DEGs identified in the CoV-2 dataset. The resulting CoV-2 network contained 272 nodes and 608 edges (Fig. S3). We additionally annotated this CoV-2 network with differential gene expression information (foldchange) identified in all infections, if present, to allow us to identify DEGs unique to CoV-2. Functionally relevant modules within this network were extracted using GLay clustering [57]. GLay is an implementation of the Girvan-Newman fast greedy community clustering algorithm. Girvan-Newman algorithm identifies communities by progressively pruning of edges to identify most densely connected clusters. Clusters with >3 nodes were retained for further analysis, resulting in a network size of 186 nodes and 9 clusters (Fig. 2). The clusters were named based on the most prominent terms associated with each cluster as identified using Enrichr [58]. All network analysis and clustering (via the clusterMaker plugin) was performed in Cytoscape [59]. Functional enrichment was performed using gene ontology (biological process) and mSigDB's Hallmark genesets (v7.1). All visualizations were generated via the ClusterProfiler library [60] available through R/Bioconductor.

## Supplementary Materials

Supplementary figures (S1–S4) and an Excel file containing supplementary tables identified in the main manuscript are included online.

Supplementary materials
