# Supplementary material for: Plasmin Cascade Mediates Thrombotic Events in SARS-CoV-2 Infection via Complement and Platelet-Activating Systems
Source: IEEE Open J Eng Med Biol. 2020 Aug 6;1:220–7. doi: 10.1109/OJEMB.2020.3014798 (PMC8527892; doi:10.1109/OJEMB.2020.3014798)
Supplement: Supplementary file 1 [file supp1-3014798.doc]

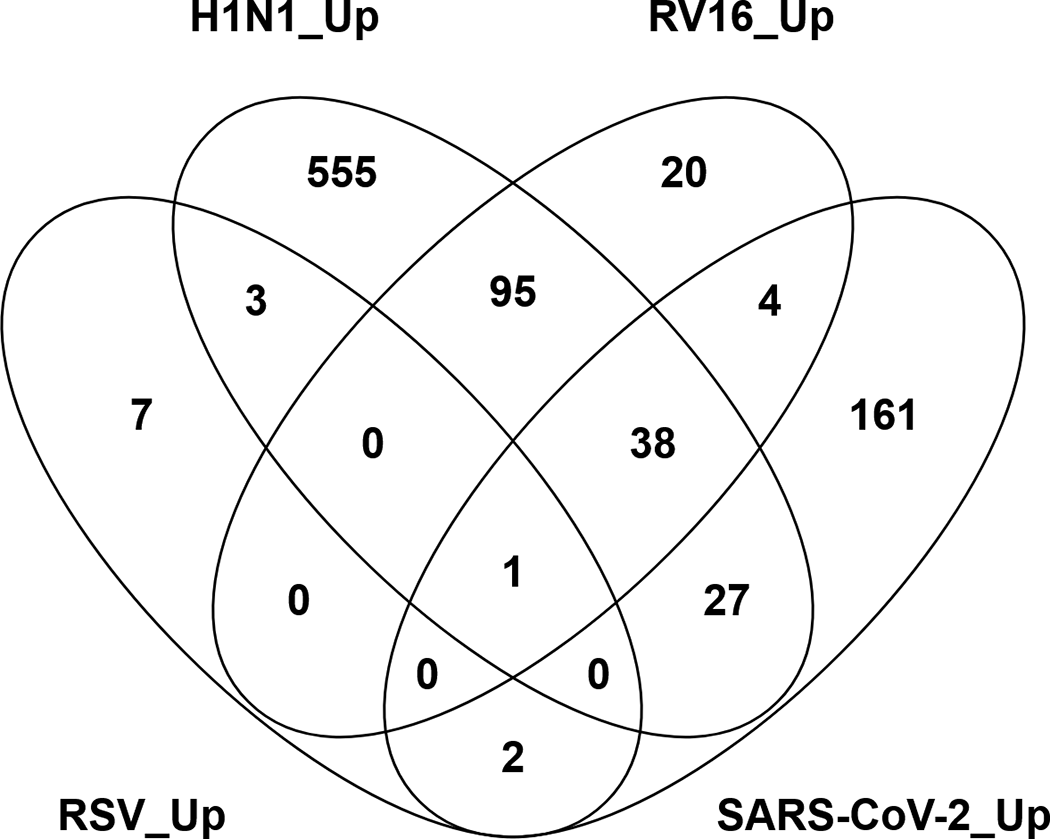


**Supplementary Materials**

# Plasmin cascade mediates thrombotic events in SARS-CoV-2 infection via complement and platelet-activating systems

**Kavitha Mukund, Kalai Mathee, and Shankar Subramaniam^*^, Member, IEEE**

Fig. S1. Overlap of upregulated gene across four upper respiratory tract infections(Rhinovirus- RV16, respiratory synytical virus RSV, and influenza H1N1) and SARS-CoV-2 (CoV-2), compared to their respective control, represented as a Venn diagram. 161 genes are uniquely differentially upregulated in SARS-CoV-2 infected cells.


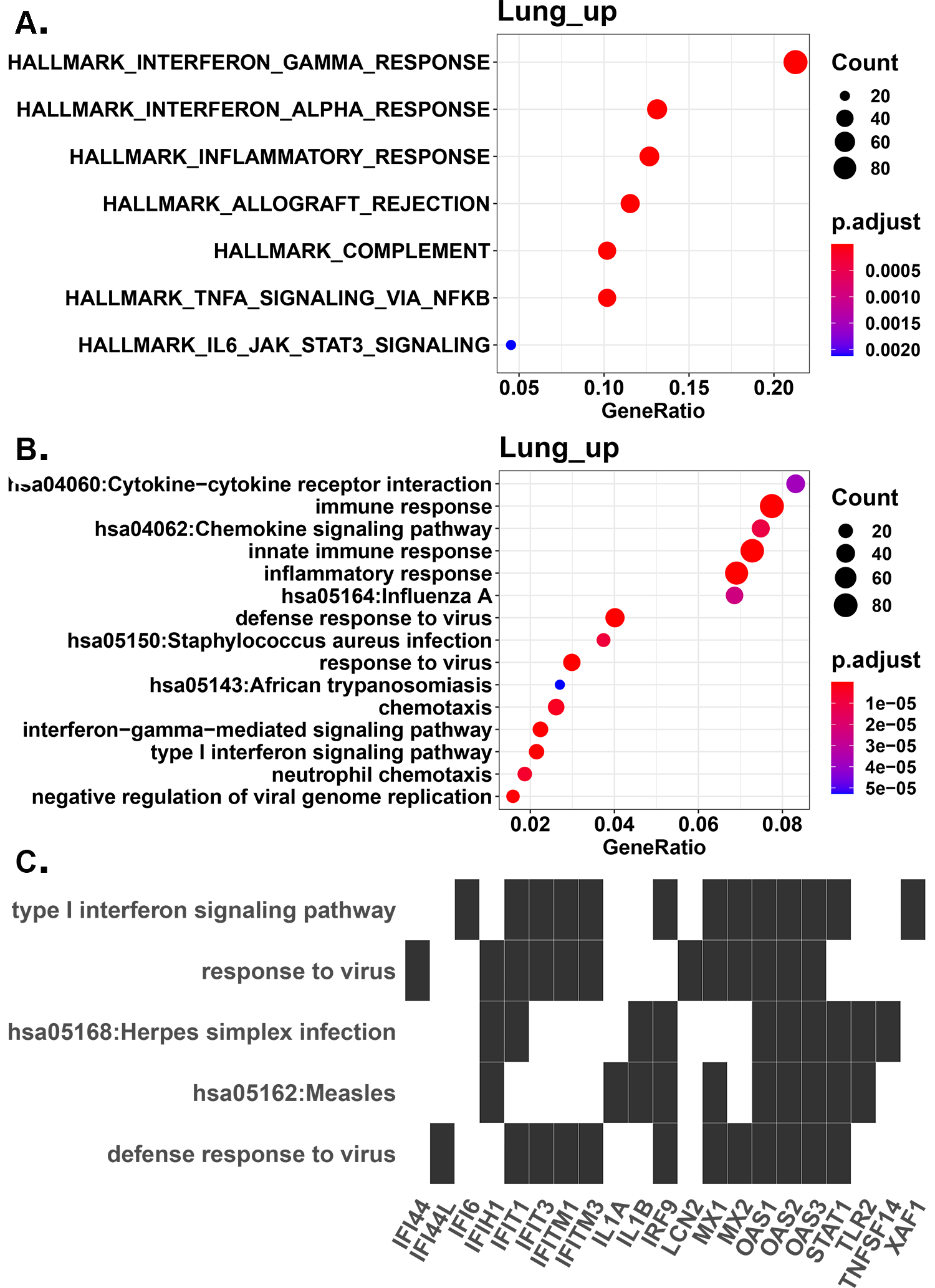


Fig. S2. **Functional analysis of differentially expressed genes (DEGs) identified in COVID lung.** Functional enrichment was identified for genes upregulated in COVID-19 lung tissue using A. mSigDB’s hallmark gene sets and B. Gene ontology’s biological process enrichment C. A heatmap plot showing the functional enrichment of genes which overlap between DEGs upregulated in CoV-2 dataset and COVID-19 lung. Number of genes within the dataset for each category are represented by the size of the dots and the p-adjusted value by the color scale indicated


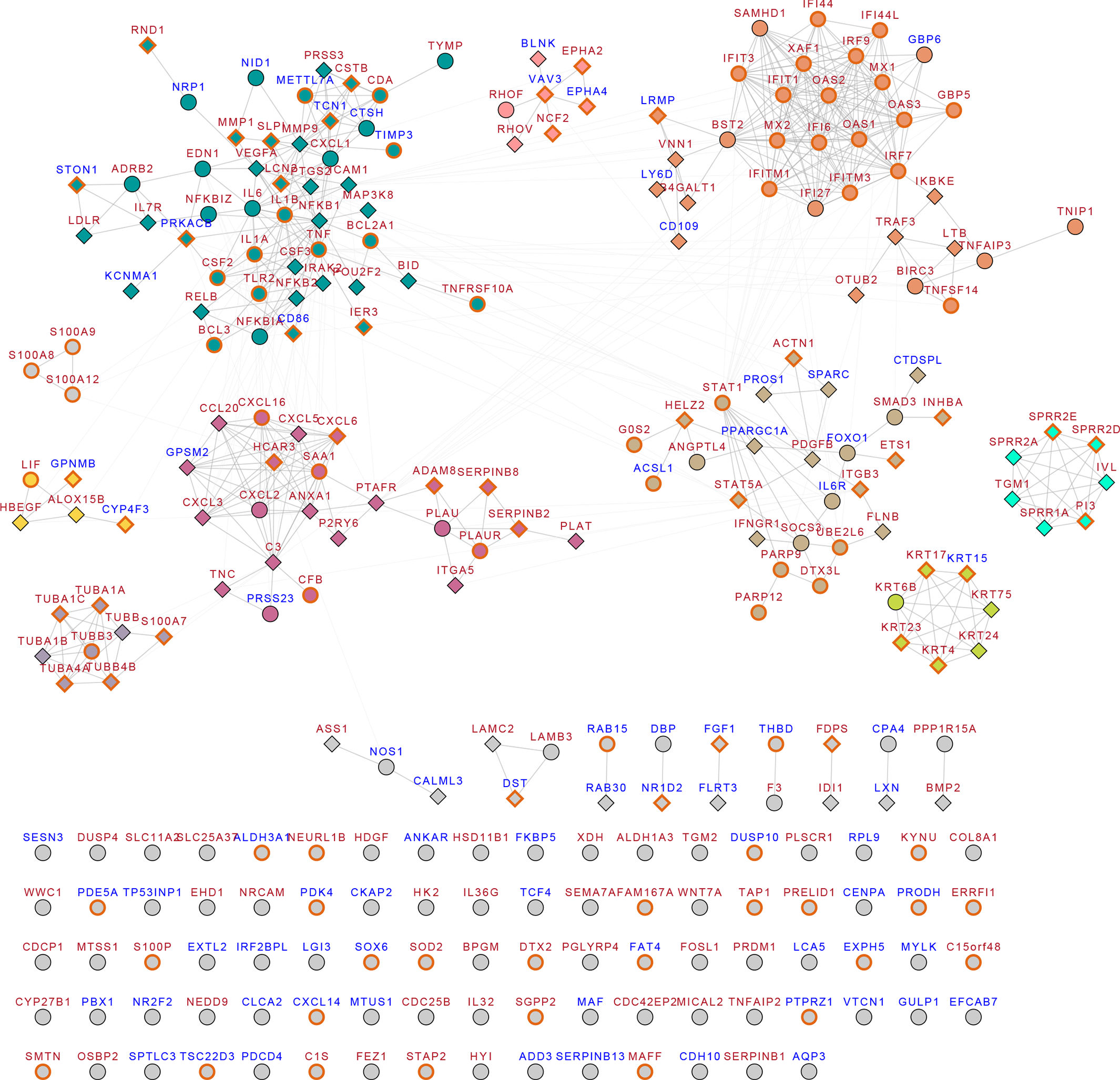


Fig. S3. **Protein interaction network for SARS-CoV-2 infection-**The entire protein-protein interaction network extracted from STRINGdb for genes differentially regulated (DEGs) in CoV-2 dataset (see Methods) is shown here. All nodes with n>3 were extracted and functionally annotated and representd in Figure 2. This figure additionally highlights genes that were identified as DEGs within the COVID-19 lung samples (indicated with an orange node border). Red node labels indicate upregulated genes and blue node labels indicates downregulated genes. Diamond node shapes indicate DEGs identified only within CoV-2 while circle indicate DEGs identified in more than one upper respiratory tract infection

.


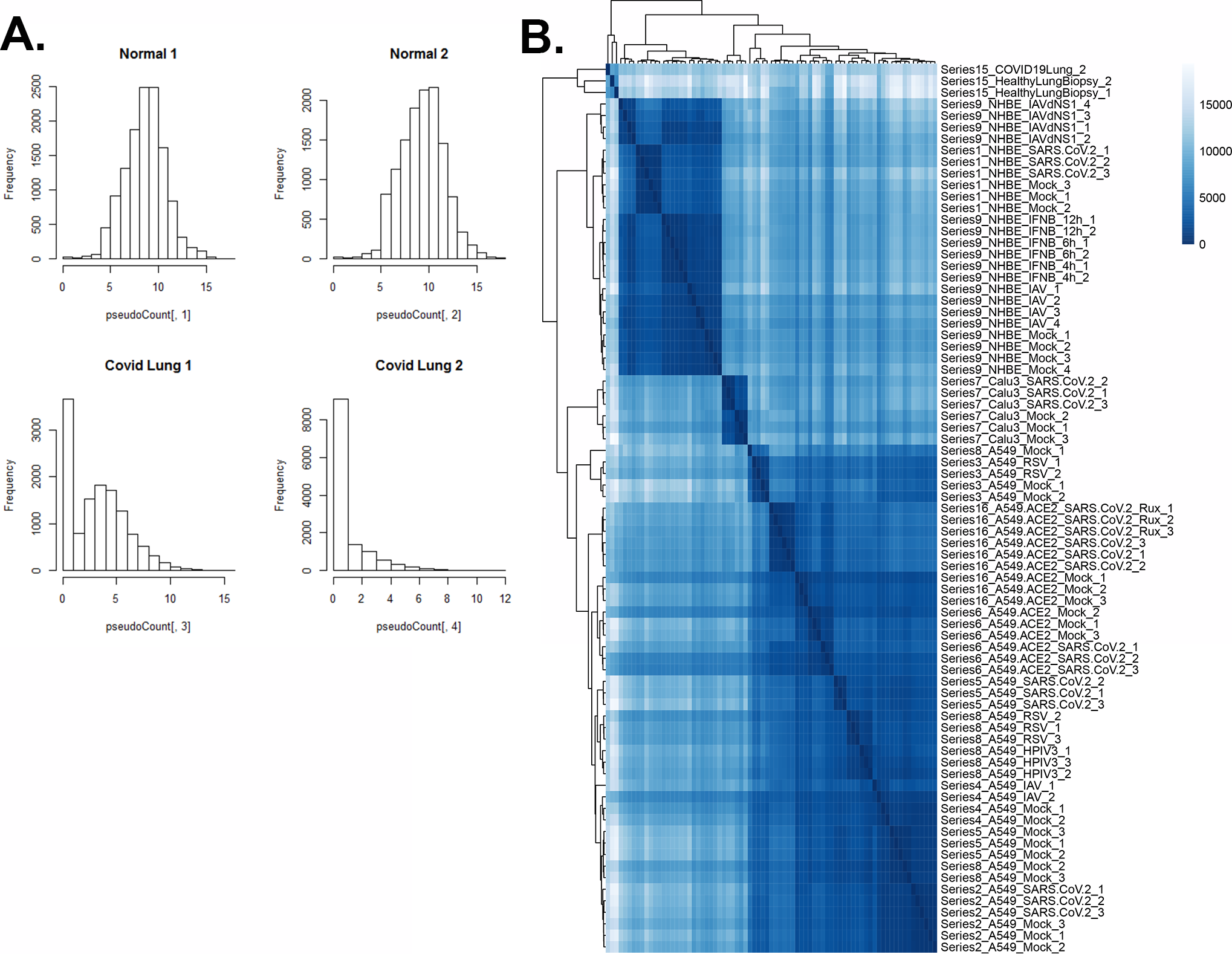


Fig. S4. Analysis of previously published SARS-CoV-2 sequencing data and rationale for using NHBE cell lines and only one COVID-19 lung tissue A. Histograms of raw counts (log 2 scale, x axis) as published in GSE145708 for biopsies obtained from normal and COVID-19 lung (n=2). These results indicate likely degradation of covid lung 2 samples. We subsequently consider this sample unusable for downstream analysis of fold changes between healthy and COVID lung B. Clustering of counts across all cell lines infected with SARS-CoV-2, Influenza (and their respective controls) and one lung biopsy as obtained from the original study. Based on the clustering tree, we reasoned that the NHBE cell line better approximates lung biopsies and used them as the primary cell line of interest for this analysis
